# The Influence of Green Product Type, Message Framing, and Anticipated Pride on Green Consumption Behavior: An Event-Related Potential (ERP) Study

**DOI:** 10.3390/brainsci13101427

**Published:** 2023-10-07

**Authors:** Guanfei Zhang, Jin Li, Min Tan, Yiping Zhong

**Affiliations:** 1 Department of Psychology, School of Education Science, Hunan Normal University, Changsha 410081, China; 18364915905@163.com (G.Z.); jimli09@163.com (J.L.); mintan2021@163.com (M.T.); 2 Cognition and Human Behavior Key Laboratory of Hunan Province, Deparment of Educational Science, Hunan Normal University, Changsha 410081, China

**Keywords:** green product type, message framing, anticipated pride, green consumption behaviors, event-related potential

## Abstract

Different types of green products require different marketing approaches to promote individual green purchasing behaviors. Previous studies have focused only on the effects of message framing on the promotion of different types of green products; however, little is known about the role of underlying emotions. Using event-related potentials (ERPs), this study investigated the neural responses to message framings and anticipated pride in green product types to assess their level of influence on green consumption. Participants in this study were randomly assigned to the anticipated pride versus control groups, and asked to make green consumption decisions involving different types (self- vs. other-interested) of green products, utilizing both gain and loss framing. The behavioral results demonstrated that participants in the anticipated pride group made more green product purchase choices than those in the control group. The ERP results showed that within the loss framing of the control group, other-interested green products induced larger N400 and smaller late positive potential (LPP) amplitudes than self-interested green products, whereas the results showed the opposite trend for the anticipated pride group. These results indicate that although individuals might have biases in their motivation that lead them to focus on self-interested green products, anticipating pride reduces cognitive conflicts and increases their motivation to focus on other-interested green products in the context of loss.

## 1. Introduction

The frequent occurrence of soil and water resource and air pollution problems has created much public concern about environmental safety and sustainable development [1]. Humans are also recognizing the significance of sustainable consumption for the health of the environment. Green consumption refers to environmentally friendly consumption that meets the needs and desires of human beings while simultaneously reducing the detrimental effects on the natural environment to a minimum [2,3,4]. This concept has received significant public attention and praise as an alternative to traditional consumption [5]. However, the very nature of green consumer behavior represents a weighing balance between personal and ecological interests [6,7,8], which demands that individuals spend their personal funds (personal interests) on green products that facilitate the achievement of environmental sustainability (ecological interests). The reality of this trade-off reduces individuals’ actual enthusiasm for green purchasing behavior [9], which means lower actual green purchasing behavior. This in turn leads to the manifestation of the most typical phenomenon regarding green consumption behavior: the intention–behavior gap [10]. Although consumers demonstrate positive intentions with regard to the consumption of green products, actual purchase behaviors are much lower [11,12,13]. Previous studies have primarily focused on exploring green consumption intentions [14,15,16]. According to the theory of planned behavior, attitude alone cannot directly and positively predict an individual’s actual behavior [17,18,19]. Therefore, for the sake of future environmental sustainability, it is vital to study effective measures that can successfully promote individuals’ green purchasing behavior.

Green consumption behavior often involves purchasing green products. Prior research has revealed that the green product type influences an individual’s green consumption behavior [3,20,21]. People’s perceived motivations for purchasing green products can be divided into two types: self-interested and other-interested green products [3,10,20]. Self-interested green products refer to products for which individuals make purchasing decisions based on personal health and other egoistic motivations, one such example being organic apples. Other-interested green products refer to products that are purchased based on altruistic motives to improve the environment, such as green batteries [3,10]. In daily life, businesses design marketing plans to promote product purchases, and the same applies to green products. And, varying types of green products require various forms of marketing programs [22]. Otherwise, if the same plan to induce purchase intention is implemented across a range of different green products, it may have a promoting effect on some, while hindering others. Previous studies have found that message framing causes a different mindset and thus promotes environmental behavior [23]: that is, message framing can have a marketing effect on environmental behavior at the cognitive level [24]. And, previous studies on actual green purchasing behavior have found that when using cognitive-level message framing as a marketing strategy for different types of green products, individuals exhibit similar actual purchasing behavior for both self-interest and other-interest green products, regardless of whether gain or loss framing is used [10]. Researchers have explained that the existence of the “intention behavior gap” means that the use of message framing alone might only affect individuals’ green purchasing intentions for different types of green products, rather than affecting their actual green purchasing behavior [10]. This indicates that using cognitive-level message framing alone is insufficient to achieve the desired marketing effects on different types of green product purchasing behaviors. According to the dual-system model (DSM) of decision-making (see Table 1 for details), both the cognitive and affective systems are effective in decision-making [25,26,27,28], where the cognitive system is contemplative and the affective system is emotional and impulsive [25]. Previous studies have found that interactions between emotions and message framings can help to shape more positive environmental attitudes in individuals [29,30], and, most importantly, emotions are an important influencing factor on the “intention–behavior gap” in ethical consumer behavior [31]. Therefore, adopting a dual marketing strategy at both the cognitive and emotional levels may be an effective approach to promote individuals’ actual green purchasing behavior.

Anticipated pride is widely used in studies on the subject of emotions to promote environmental behaviors [32,33]. Pride is a positive self-conscious emotion that arises in relation to the self in order to achieve socially desired goals [34,35]. Anticipated emotions refer to emotional sensations generated by people’s predictions of possible future outcomes [36,37]. It has previously been found that anticipated pride can significantly increase individuals’ green purchase intentions [38,39]. Most importantly, anticipated pride moderates the effects of green product type and message framing on green consumption decisions, respectively. In previous studies on the joint impact of green product types and anticipated pride on green consumption behavior, there is indirect evidence that anticipated pride is more related to altruistic than self-interest motivation. First, pride is a self-awareness emotion that arises after accomplishing something perceived as valuable or virtuous [40,41,42], and can motivate individuals to behave, in the future, in accordance with socially valued standards [43]. Other-interested green products have purely altruistic motives for protecting the environment, whereas self-interested green products have more self-oriented or selfish connotations [44]. Thus, the former is more socially recognized and aligned with social norms than the latter [45]. Therefore, anticipated pride is expected to promote individual green purchasing behavior when paired with other-interested green products that are more aligned with social value standards than self-interested green products. Further, Onwezen et al. [46] also found that the promoting effect of anticipated pride was more evident in fair-trade consumption related to altruistic motives than in organic food consumption related to personal motives. This further affirms the notion that anticipated pride is more likely to have a promoting effect on other-interested green products with altruistic motives than on self-interested green products with egoistic motives.

Previous studies regarding the effects of anticipated pride and message framing on green consumption have found that individuals motivated by anticipated pride were more inclined to environmentally friendly behaviors than individuals induced to do so by anticipated guilt through gain framing, manifested as making more financial donations to environmental organizations [47]. According to the dual-process model of approach and avoidance motivation [48], there are two motivational systems that guide people’s behavior (see Table 1 for details). One is the behavioral approach system (BAS), under which individuals are motivated to approach positive cues such as reward as well as non-punishment, and the other is the behavioral inhibition system (BIS), under which individuals are motivated to avert and avoid negative cues such as punishment [29,49]. Emotions and motivation are closely linked and intertwined [50], with emotions activating an individual’s motivation to act and inducing corresponding behaviors in certain situations [51]. Anticipated pride is a positive, self-conscious emotion [34,35] that activates an individual’s behavioral approach system and demonstrates a search for positive outcomes. Gain framing emphasizes the benefits that come from protecting the environment, which is the same motivation for approaching the anticipated pride, which produces a facilitative effect on green consumption behavior.

**Table 1:** Comparison of Two Models.

Model Name	Model Content	Features
Dual-system model (DSM)	It involves both cognitive and affective systems [25,26,27,28]. The cognitive system is contemplative, flexible, slow and strategic, centered on self-regulation and self-control, whereas the affective system is emotional, intuitive, fast and impulsive [25]. Both help individuals to make decisions.	Related to decision-making, includes cognitive and affective systems.
Dual-process model of approach and avoidance motivation	It has two motivational systems: the behavioral approach system (BAS) and the behavioral inhibition system (BIS). The BAS allows individuals to show approach motivation to positive cues such as rewards, non-punishment, and avoidance of punishment. The BIS causes individuals to develop avoidance motivation to avoid punishment [29,48,49].	Related to behavioral motivation, includes BAS and BIS, inextricably linked to emotions. Approach motivation is associated with positive emotions. Avoidance motivation is associated with negative emotions.

In summary, anticipated pride moderates the impact of message framing and green product types on green consumption behavior, respectively. However, to date, no research has explored the impact of both cognitive-level message framing and affective-level anticipated pride on purchase decision-making for varying types of green products. Therefore, exploring the roles of all three phenomena in green consumption can help people to better understand the neural mechanisms behind green purchasing decisions and effective methods used for promotion. Based on the above analysis, the present study set up a priming (the anticipated pride group) versus non-priming (the control group) anticipated pride condition on the affective level and a gain versus loss framing on the cognitive level, and so the following hypothesis is proposed:

**Hypothesis 1 (H1).**  *Compared to the control group, individuals will engage in more green purchasing behaviors when gain framing rather than loss framing is combined with other-interested green products under the anticipated pride group*.

Previous studies have focused on measuring individuals’ willingness to engage in green consumption through questionnaires [14,15,16]. However, the existence of social approval bias may lead to a separation between individual explicit subjective reporting and actual intrinsic neural responses [10,52]. Previous studies have explored this using functional magnetic resonance imaging (fMRI) and found that while individuals verbally reported being more interested in green advertisements, fMRI results revealed stronger activation in brain areas related to personal value assessments and rewards (ventromedial prefrontal cortex and ventral striatum) for the control group of advertisements compared to green advertisements [53]. This partly accounts for the separation of individual subjective reports from intrinsic neural mechanisms in terms of brain area activation, but whether there is a separation between the two in terms of time course is still unknown. Neuroscientific techniques characterized by high temporal resolution are event-related potentials (ERPs), which allow for measuring the time course and potential neural mechanisms involved in brain activity when individuals make social decisions [47,54]. Previous studies on the ERP for green consumption decisions have investigated three ERP components: P3, N400, and late positive potential (LPP) [10,55,56]. The P3 is a positive wave that peaks 300–500 ms after stimulus onset and has been linked to the allocation of cognitive resources in decision-making [10,57,58,59,60]. The higher the correlation with self, the stronger the individual’s motivation and the more cognitive resources they invest, resulting in a larger amplitude of P3 amplitudes [10]. It was found that individuals invested more cognitive resources and evoked greater P3 amplitudes when making green purchase decisions for friends with high self-relevance compared to strangers with low self-relevance under the non-observe condition [58]. The N400 is a type of negative wave that peaks at around 400 ms after stimulus onset, and is an indicator of cognitive and emotional conflict [61,62,63]. It has been previously observed that compared to the control group, individuals who induce empathy with nature will reduce their cognitive and emotional conflicts when purchasing green products, manifested as smaller N400 amplitudes [55]. The LPP is a long-duration positive wave that emerges roughly 300–500 ms following stimulus onset [64,65] and reflects the strength of motivation directed toward an emotional stimulus [66,67,68]. Research has found that individuals have stronger emotional motivation towards the loss framing rather than the gain framing when making green consumption willingness decisions, as evidenced by larger LPP amplitudes [56].

As mentioned in the introduction section, a prediction of this study is that under different anticipated pride emotion groups, different types of message framing acting on distinct types of green products will trigger diverse neurological reactions to green consumption behavior, as indicated by P3, N400, and LPP. First, P3 is linked to the cognitive resource investment of individuals in decision-making; the more cognitive resources an individual invests, the greater the P3 amplitude [10,57,58,59,60]. Previous studies have found that when emphasizing gain, individuals invest more cognitive resources in people and things related to themselves, while investing similar cognitive resources in things related to themselves and others (the environment) when losses occur [10,57]. Therefore, the following hypothesis is proposed:

**Hypothesis 2a (H2a).**  *In the control group, self-interested green products will induce greater P3 amplitudes than other-interested green products through gain framing rather than loss framing*.

Pride can promote individuals’ pursuits of valuable goal behaviors [69] and altruism [42,70,71]. Accordingly, it is anticipated that pride can promote altruistic motivation for individuals. Therefore, corresponding to H2a, the following hypothesis is proposed:

**Hypothesis 2b (H2b).**  *In the anticipated pride group, other-interested green products will have greater P3 amplitudes than self-interested green products for gain framing rather than loss framing*.

Second, N400 is associated with cognitive and emotional conflicts in social decision-making, such as violations of social norms [72] or personal value systems [73], resulting in a greater N400 effect. Therefore, when the description “purchasing self-interested (other-interested) green products brings health (environmental) benefits” under gain framing and “not purchasing self-interested green products brings harm to physical health” under the loss framing is compared to “not purchasing other-interested green products brings environmental losses” under loss framing, the loss framing which causes damage to the environment further violates the social norms of protecting the environment that individuals accept [10]. Thus, loss framing that causes harm to the environment induces stronger cognitive conflicts and negative emotions in individuals. Therefore, the following hypothesis is proposed:

**Hypothesis 3a (H3a).**  *In the control group, other-interested green products induce greater N400 amplitudes than self-interested green products through loss framing rather than gain framing*.

According to the above projection, anticipated pride has a stronger promoting effect on altruistic motivation than it does on self-interest [42,46,70]. Therefore, people tend to form a value system in which pride is linked to social interests rather than self-interest. Thus, people perceive that self-inaction leads to impaired social interest rather than impaired self-interest, which causes them to feel less pride. In this instance, the description of “not purchasing other-interested green products brings environmental losses and makes people feel less pride” is more consistent with cognitive logic. Thus, corresponding to H3a, the following hypothesis is proposed:

**Hypothesis 3b (H3b).**  *In the anticipated pride group, other-interest green products induce smaller N400 amplitudes than self-interested green products through loss framing rather than gain framing*.

Finally, LPP reflects an individual’s emotional and motivational arousal in response to stimuli, and the resulting allocation of attention [66,67,68]. Previous studies have found that LPP has a bias toward self-interest loss [67,74]. This means that individuals have stronger motivation and emotional arousal when they experience a loss of self over others, as compared to a gain of self over others [67], or a joint loss of self and others [74]. Thus, a loss of self over others will induce larger LPP amplitudes [67]. This also suggests that LPP is highly sensitive to the loss of self-interest. Therefore, the following hypothesis is proposed:

**Hypothesis 4a (H4a).**  *In the control group, self-interested green products will produce larger LPP amplitudes than other-interested green products through loss framing rather than gain framing*.

Anticipated pride can significantly increase individuals’ altruistic motivation, thus increasing their concern for environmental benefits, reversing self-interest loss bias, or equalizing it with self-interest concerns. Thus, the following hypothesis is proposed:

**Hypothesis 4b (H4b).**  *In the anticipated pride group, other-interested green products will have similar or greater LPP amplitudes than self-interested green products through loss framing rather than gain framing*.

## 2. Materials and Methods

### 2.1. Participants and Experimental Design

We employed a 2 (green product type: self-interested vs. other-interested) × 2 (message framing: gain vs. loss) × 2 (anticipated pride: anticipated pride group vs. control group) mixed experimental design, where the first two independent variables were within-subjects variables while the third variable was between-subjects [10,56,75,76]. We performed a priori power analysis using MorePower 6.0 to calculate the minimum sample size [77]: η_p_^2^ = 0.12 for interaction between three variables [78], power = 0.8, α = 0.05. The results suggested that 30 participants in each group would ensure 80% statistical power. Sixty undergraduate or graduate students (mean age = 20.27, standard deviation (SD) = 1.74, range = 18–26 years) from Hunan Normal University participated in this experiment, including 30 participants in the anticipated pride group (18 female and 12 male) and 30 participants in the control group (18 female and 12 male). According to the previous literature P3, LPP, and N400 should have a minimum trial of 40 trials and above [79,80,81], and since each of our participants was able to meet this criterion in terms of the number of trials in each condition (see Table S1), no participants were excluded. Participants involved in this study were of right-handedness, had standard or corrected standard vision, were not color-blind, had no history of psychiatric illness, and signed an informed consent form before the experiment was formally conducted. Depending on the product purchases made by the participants in the experimental task (common products vs. green products), rewards were given at the end of the experiment. That is, the cost of the products purchased by the participants was deducted from the CNY 50 purchase fund, and the remaining cost was the final reward received by the participants (see Section 2.2.4 for details).

### 2.2. Experimental Materials

#### 2.2.1. Green Product Type Manipulation

Consistent with the manipulation of green product types in Zhang, Li, Li, Tan, Li, and Zhong [10]’s article, the names and pictures of a total of 18 green products (nine self- vs. other-interested green products) were used as starter materials. There was no significant difference in familiarity (*M*_self-interested_ = 6.06 ± 1.30, *M*_other-interested_ = 5.50 ± 1.20, *t* (58) = 1.709, *p* = 0.093), arousal (*M*_self-interested_ = 5.89 ± 1.16, *M*_other-interested_ = 5.73 ± 0.80, *t* (58) = 0.632, *p* = 0.530), and product preference (*M*_self-interested_ = 6.48 ± 1.10, *M*_other-interested_ = 6.28 ± 1.04, *t* (58) = 0.724, *p* = 0.472) between the selected experimental materials of self- and other-interest green products [10]. When evaluating the self-interest and other-interest attributes of green products, the results showed that the selected products could represent the type of green products they belong to well [10], see Table S2 for details.

#### 2.2.2. Message Framing Manipulation

Referring to previous studies [10,29,82,83,84], the specific manipulation of the message framing was as follows: “Buy green products, and the environment (you) obtains water and soil (health)” (gain framing) and “Do not buy green products, and the environment (you) loses soil and water (health)” (loss framing).

#### 2.2.3. Anticipated Pride Manipulation

The manipulation of the anticipated pride was adapted based on Zubair, Iqbal, Usman, Awais, Wang and Wang [56], and Schneider, Zaval, Weber and Markowitz [38]. The anticipated pride manipulation was “I will be proud” (under the context of gain message) or “I will not be proud” (under the context of loss message), and the control group manipulation was manipulated as a black blank screen with the same duration as the anticipated pride group.

#### 2.2.4. The Dilemma of the Environmental Decision Paradigm

Green consumption behavior is a balance between one’s own benefits and those of the environment [6,7,8]; so, we used the environmental dilemma decision-making paradigm to measure the actual green purchase behavior of individuals [10,58,85]. In this paradigm, common products and their prices were on the left side of the decision-making interface, and green products and their prices were on the right side [86]. In order to build a dilemma conflict scenario and conform to the reality of real life, the price of green products was always set higher than the price of common products. And, in order to prevent the extreme situation that the participant kept pressing a key, according to this paradigm, the prices of green products were set to be 25%, 50%, 75%, 100%, 125%, 150%, and 175% higher than the prices of common products [10,58,85,87]. These seven price–cost differences would not affect our main variables [10]. During the experiment, the participants were given a purchase fund of CNY 50 for multiple rounds of purchase, and finally a random round of purchase decisions was selected and the price of the purchased products was deducted with the CNY 50 fund. The remaining part was the final payment of the participants [85]. Therefore, the purchase of green products meant less test fees (environmental benefits first), while the purchase of common products meant higher test fees (self-interest first). This was to examine whether the participants exhibited real green purchase behavior. This paradigm has been applied several times in the field of green consumption decision-making and has been well evaluated [10,55,58,85].

### 2.3. Experimental Procedure

**Figure 1:**
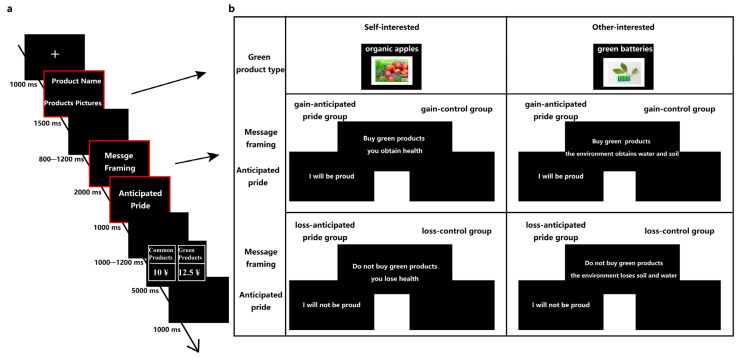
A single trial description of the experimental procedure. Note: For the part marked with red square in procedure (**a**), see (**b**) for the specific operation of each experimental condition.

In the formal experimental task, participants would be asked to sit in a chair comfortably with a distance of approximately 75 cm between their eyes and the computer screen. As depicted in Figure 1, for this experiment, on each trial, participants were first presented with a 1000 ms “+” fixation point, which signaled the official start of the experiment. Then, a green product name (at the top of the screen) and a picture (at the bottom of the screen) were presented for 1500 ms. After this, the manipulated sentences of the message framing were presented for 2000 ms at random intervals of 800–1200 ms. The following was the manipulation interface of anticipated pride (anticipated pride statement or black blank screen of the same time), with a rendering time of 1000 ms. After presenting a blank black screen with a random time interval of 1000–1200 ms, the product purchase decision interface appeared, with a presentation time of 5000 ms. Participants needed to make a key response on this interface, requiring them to make a key decision as soon as possible within 5000 ms. After the participants made the key decision, they would present a 1000 ms black blank screen, and then start the next trial. The total number of trials was 252. It was expected that there were 4 experimental conditions in the anticipated pride group and the control group, and there were 63 trials in each experimental condition. There were 4 blocks in the whole experiment, and participants would have a rest between each block.

The task of the experiment was to ask participants to watch the green products presented in the experiment, and to ask them to clarify what green products they bought in each trial and what type they belonged to. Then, they watched the message framing and the manipulation statement of the anticipated pride interface, and made the purchase decision according to the feeling after reading. The purchase decision interface required participants to make a key response as soon as possible within 5000 ms. If they wanted to buy a common product, they press the “F” key, and if they wanted to buy green products, they pressed the “J” key. If the button was not pressed for over 5000 ms, it would be considered unresponsive. Prior to the experiments formally, participants would be asked to practice to become familiar with the procedure. Participants would be given sufficient time to become familiar with the relevant information of green products (name, picture, and type of green products) before the formal experiment.

### 2.4. Electroencephalogram (EEG) Recording and Analysis

A continuous electroencephalogram was recorded using 64 Ag/AgCl electrodes cap extended by the international 10–20 system (ANT Neuro system), using a band-pass filter of 0.01–100 Hz, and continuously sampled at 500 Hz/channel. Meanwhile, CPz was used as an online reference for all electrodes, and the mean values of the left and right mastoid electrodes were used offline as a re-reference. The experiments were carried out to ensure that all electrode point resistances were below 5 kΩ. We used MATLAB 2015a and the EEGLAB toolbox (v.13.4.4 b) to pre-process and analyze the EEG data. Using a 0.1–30 Hz digital band-pass filter, we filtered the EEG data, and using independent component analysis (ICA), we removed eye blinks and vertical eye movements [88,89]. Then, the EEG data were segmented into stimulus-locked epochs ranging from 200 ms before the purchase decision statement to 800 ms afterward. After baseline correction, epochs contaminated by significant artifacts over ±75 μV were eliminated from onward analysis. Then, epochs were respectively averaged for eight conditions of each participant.

**Figure 2:**
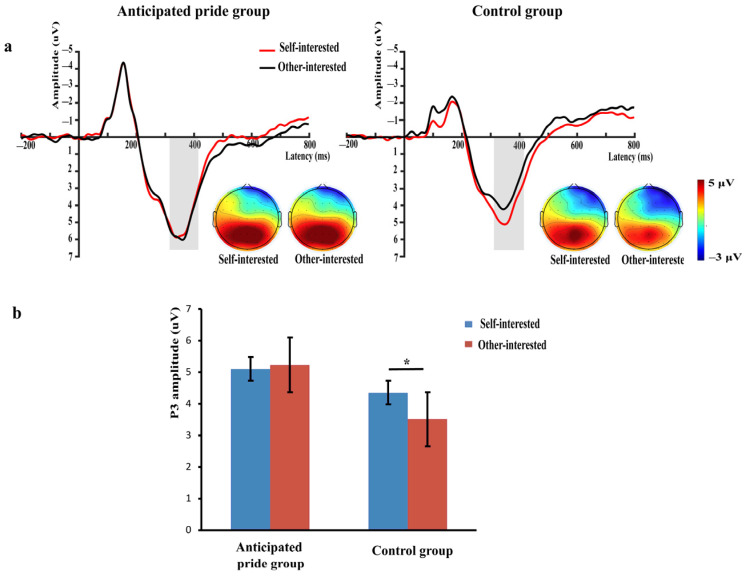
(**a**) Grand-average ERP waveforms of the six selected electrodes (CP3, CPz, CP4, P3, Pz, and P4). The grey bars highlight the time window of P3 (315–415 ms); topographies voltage distribution of P3 for four conditions (green product type × anticipated pride); (**b**) the bar graphs of mean P3 values for four conditions. * *p* < 0.05.

**Figure 3:**
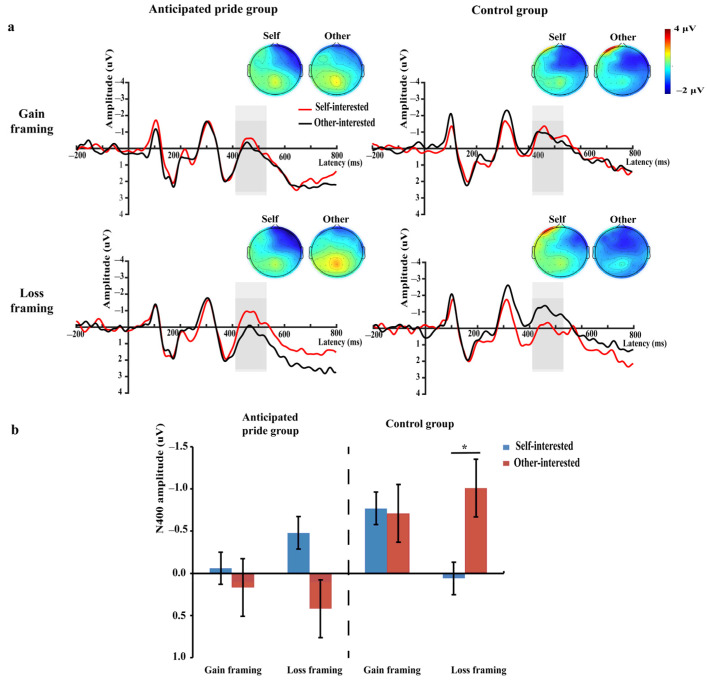
(**a**) Grand-average ERP waveforms of the six selected electrodes (F1, Fz, F2, FC1, FCz, and FC2). The grey bars highlight the time window of N400 (410–530 ms); topographies voltage distribution of N400 for eight conditions; (**b**) the bar graphs of mean N400 values for eight conditions. * *p* < 0.05. Self: self-interested green products; other: other-interested green products.

**Figure 4:**
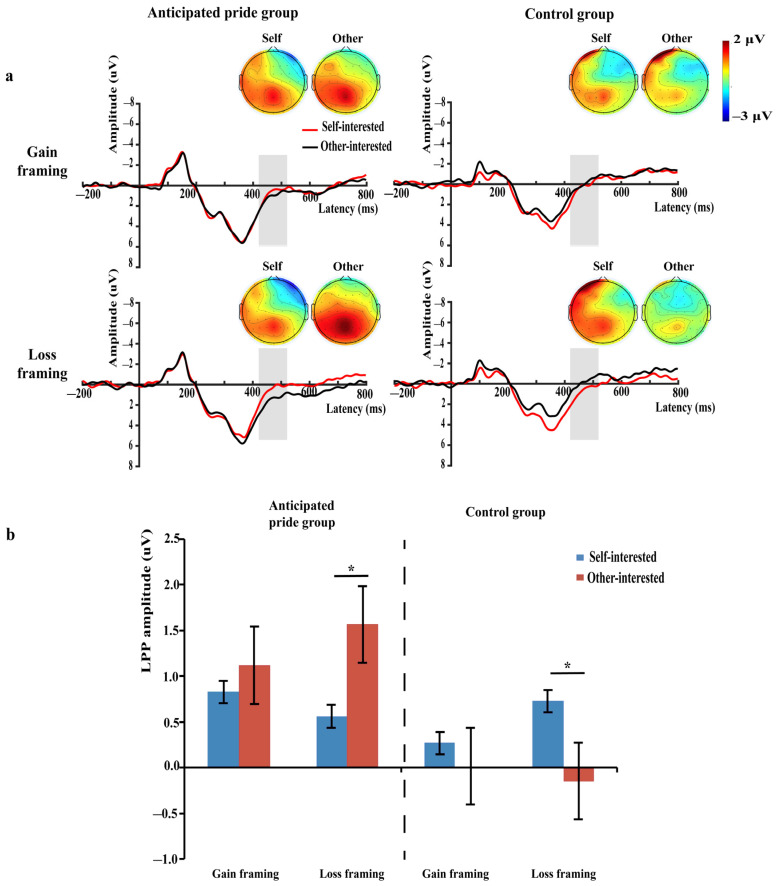
(**a**) Grand-average ERP waveforms of the nine selected electrodes (C1, Cz, C2, CP1, CPz, CP2, P1, Pz, and P2). The grey bars highlight the time window of LPP (420–520 ms); topographies voltage distribution of LPP for eight conditions; (**b**) the bar graphs of mean LPP values for eight conditions. * *p* < 0.05.

Based on visible examination of the grand-averaged ERPs and prior studies [10,55,56], we analyzed average amplitudes of the P3 (315–415 ms), N400 (410–530 ms), and LPP (420–520 ms). Based on previous studies and topographic map distributions (Figure 2a, Figure 3a and Figure 4a), we selected six electrode sites located in the central-parietal region to calculate the average amplitude of the P3 component (CP3, CPz, CP4, P3, Pz, and P4) [10,54]; six electrode sites located in the fronto-central region to calculate the mean amplitude of the N400 component (F1, Fz, F2, FC1, FCz, and FC2) [55,63]; and nine electrode sites in the central-parietal region to calculate the average amplitude of LPP component (C1, Cz, C2, CP1, CPz, CP2, P1, Pz, and P2) [47,56]. Statistical analysis was performed using SPSS 26.0, and both behavioral and EEG data were analyzed using a 2 (green product types: self-interested vs. other-interested) × 2 (message framing: gain vs. loss) × 2 (anticipated pride: anticipated pride group vs. control group) mixed repeated-measures analysis of variance (ANOVA). Using the Greenhouse–Geisser correction accounted for sphericity violations. The significance level of statistical differences was established at *p* < 0.05 and the partial eta-squared (η_p_^2^) was used as the reported indicator of the measure of effect size.

## 3. Results

### 3.1. Behavioral Results

**Figure 5:**
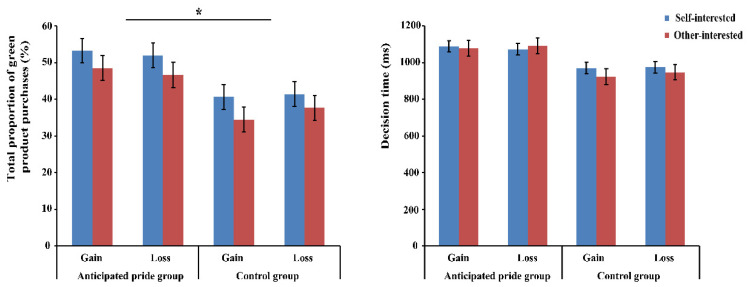
Bar graph of the total proportion of green product purchases (**left**) and the decision time (**right**) in eight conditions. * *p* < 0.05. Self-interested: self-interested green products; other-interested: other-interested green products. Gain: gain framing; loss: loss framing.

The total proportion of purchasing green products was set as the ratio of the number of times green products were purchased to the total number of decisions made [10], as shown in Figure 5. A repeated measure analysis of variance (ANOVA) of 2 (green product type: self-interested vs. other-interested) × 2 (message framing: gain vs. loss) × 2 (anticipated pride: anticipated pride group vs. control group) was conducted for the purchase rate of green products. Anticipated pride had a significant main effect, *F* (1, 59) = 4.04, *p* = 0.049, η_p_^2^ = 0.07, showing that the participants were more inclined to buy green products in the anticipated pride group (50.14%) than in the control group (38.56%). There was a non-significant main effect of green product type, *F* (1, 59) = 3.46, *p* = 0.068. The two-way interaction between message framing and anticipated pride was also statistically insignificant, *F* (1, 59) = 3.00, *p* = 0.089. No other significant impacts were identified (*ps* > 0.05).

Finally, we examined the decision times. Neither the main effect nor the interaction achieved significance (all *p*-values > 0.05).

### 3.2. ERP Results

#### 3.2.1. P3 (315–415 ms)

A significant two-way interaction was found between green product type × anticipated pride, *F* (1, 58) = 4.60, *p =* 0.036, η_p_^2^ = 0.07. A simple effects analysis was performed next to explore this interaction. In the control group, the P3 was more positive regarding the green products of self-interest (4.31 ± 3.64 μV) compared to the other-interested green products (3.45 ± 3.37 μV), *F* (1, 58) = 6.83, *p* = 0.011, η_p_^2^ = 0.11, but there was no difference between self-interested (5.10 ± 2.44 μV) and other-interested green products (5.24 ± 2.59 μV) in the anticipated pride group, *F* (1, 58) = 0.18, *p* = 0.677 (see Figure 2). No other significant impacts were identified (*ps* > 0.05).

#### 3.2.2. N400 (410–530 ms)

A significant three-way interaction was found between green product type × message framing × anticipated pride, *F* (1, 58) = 4.98, *p* = 0.030, η_p_^2^ = 0.08. To investigate this interaction, we performed a simple effect analysis to examine this interaction. In the anticipated pride group, the results suggested that the N400 was non-significant among self-interested and other-interested green products at the loss framing (*M_self-interested_* = −0.48 μV vs. *M_other-interested_* = 0.42 μV), *F_loss_* (1, 58) = 3.29, *p* = 0.075, and the N400 also suggested no difference between self- and other-interested green products at the gain framing (*M_self-interested_* = −0.06 μV vs. *M_other-interested_* = 0.17 μV), *F_gain_* (1, 58) = 0.18, *p* = 0.671. While in the control group, the outcomes showed that the N400 has a greater negative amplitude in the other-interested than in the self-interested green products under loss framing (*M_self-interested_* = 0.06 μV vs. *M_other-interested_* = −1.01 μV), *F_loss_* (1, 58) = 4.60, *p* = 0.036, η_p_^2^ = 0.07, but the N400 had no difference among self- and other-interested green products under gain framing (*M_self-interested_* = −0.77 μV vs. *M_other-interested_* = −0.71 μV), *F_gain_* (1, 58) = 0.01, *p* = 0.903 (see Figure 3). No other significant impacts were identified (*ps* > 0.05).

#### 3.2.3. LPP (420–520 ms)

A repeated-measures ANOVA of green product type × anticipated pride showed a significant two-way interaction effect, *F* (1, 58) = 5.26, *p* = 0.025, η_p_^2^ = 0.08. A simple effect analysis was performed to evaluate this interaction. However, the LPP was nonsignificant between other-interested (1.34 ± 3.53 μV) and self-interested green products (0.69 ± 2.63 μV) in the anticipated pride group, *F* (1, 58) = 2.99, *p* = 0.089, and the LPP also suggested no difference among self- (0.50 ± 2.86 μV) and other-interested green products (−0.07 ± 3.15 μV) in the control group, *F* (1, 58) = 2.29, *p* = 0.136.

More importantly, we found a significant three-way interaction between green product type, message framing, and anticipated pride, *F* (1, 58) = 4.03, *p* = 0.049, η_p_^2^ = 0.07. We performed a simple effect analysis to explore this significant interaction. In the anticipated pride group, the results showed that the LPP had a greater positive amplitude on the other-interested than on the self-interested green products under loss framing (*M_self-interested_* = 0.56 μV vs. *M_other-interested_* = 1.57 μV), *F_loss_* (1, 58) = 5.57, *p* = 0.022, η_p_^2^ = 0.09, but the positive amplitude of LPP indicated no difference among self- and other-interested green products under gain framing (*M_self-interested_* = 1.12 μV vs. *M_other-interested_* = 0.83 μV), *F_gain_* (1, 58) = 0.39, *p* = 0.535. In contrast, in the control group, the outcomes showed that the LPP had a greater positive amplitude on the self-interested than on the other-interested green products under loss framing (*M_self-interested_* = 0.73 μV vs. *M_other-interested_* = −0.16 μV), *F_loss_* (1, 58) = 4.21, *p* = 0.045, η_p_^2^ = 0.07, but the positive amplitude of LPP indicated no difference among self- and other-interested green products under gain framing (*M_self-interested_* = 0.27 μV vs. *M_other-interested_* = 0.01 μV), *F_gain_* (1, 58) = 0.31, *p* = 0.580 (see Figure 4). No other significant impacts were identified (*ps* > 0.05).

## 4. Discussion

This study used a high temporal resolution neurophysiological approach to investigate how anticipated pride and message framing modulate the effects of green product types in green consumption. Behavioral results showed that individuals chose more green purchases under anticipated pride compared to the control group. In terms of the ERPs results, anticipated pride modulated the effect of green product type on green consumption behavior, as manifested in the P3 components. Most importantly, anticipated pride was found to moderate the effects of green product types in green consumption behavior, in conjunction with message framing, as reflected by N400 and LPP.

### 4.1. Green Consumption Behavior Is Influenced by Anticipated Pride

In this study, we had not observed any interaction between green product type, message framing, and anticipated pride in behavioral outcomes. This failed to validate H1, and also this failed to validate the dual-process model of approach and avoidance motivation. This may be attributable to the discrepancy that exists between green consumption intentions and actual green consumption behaviors [11,12,13]. Previous studies have measured green consumption intentions rather than the actual behavior, which was used in our study. In exploring the intention of green consumption, there is no need for individuals to spend actual monetary costs, only to express their own attitude. This also means that there will be social approval effects. And, in the actual green purchasing behavior, individuals need to realistically spend the monetary expense of self to contribute to environmental friendliness, which reduces the influence of social desirability to a certain extent. However, this also weakens the facilitating effect of both anticipated pride and message framing in actual green purchasing behavior, and thus this resulted in the absence of the expected hypothesis [10]. However, we observed that compared to the control group, individuals engaged in more green purchasing behavior under the condition of anticipated pride, indicating a positive promoting effect for anticipated pride. This discovery is in accordance with the results of prior studies [32,38]. Green consumer behavior involves weighing the balance of personal and ecological interests [6,7,8], which requires individuals to exercise behavioral control to overcome their selfish desires and make more environmentally friendly choices for the future [90,91]. Moreover, anticipated pride enhances behavioral inhibition control [70,92] and delays gratification [93]. This satisfies the basic need for individuals to engage in green consumption behaviors; thus, anticipated pride exhibited a positive facilitative effect in this study. Meanwhile, the positive aspects of mood have recently received attention from researchers. During the experimental process, each participant in the anticipated pride group had to undergo multiple consecutive emotional primes, which could generate a relatively sustained and positive “warm glow” good feeling [94,95], indicating that the individual entered a sustained and positive mood state in the experiment. Previous studies have found that mood is related to individuals’ self-control and pursuit of goals [96,97]. For example, basic positive mood (e.g., happiness) is related to the achievement of short-term goals, while high-level positive mood (e.g., anticipated pride) can promote individuals’ pursuit of long-term goals [96]. This is manifested in this study as making more behaviors that are conducive to long-term sustainable goals (green purchasing behavior). It could also mean that this emotional priming may resemble behavioral and cognitive neural patterns triggered by mood, which requires further research in the future to provide direct evidence. This could provide some ref-erences or possibilities for future research on the positive aspects of mood.

### 4.2. The P3 Is Modulated by Green Product Type and Anticipated Pride

A joint effect of green product type, message framing, and anticipated pride on the P3 component was not observed at the time of decision-making, a finding that is inconsistent with both H2a and H2b. Interestingly, a moderating effect of anticipated pride was observed on the P3 components of green product types. Specifically, in the control group, individuals had a greater P3 when making decisions for self-interested green products rather than other-interested green products. Conversely, in the anticipated pride group, the difference in P3 between self- and other-interested green products disappeared.

P3 represents the cognitive resources invested by individuals in social decision-making; the larger the amplitude of P3, the more cognitive resources invested in decision-making [57,59]. Thus, our findings suggested that individuals are inherently egoistic, but that anticipated pride can potentially increase the amount of attention paid to altruistic motives. This was reflected in the fact that while individuals invested more cognitive resources in green products related to self-interest, the addition of anticipated pride could help to shift individuals’ cognitive resources. This shows that participants invested the same degree of cognitive resources in environmental benefits as in self-interest. It is congruent with the findings of earlier research, as described below. People tend to maximize their own interests rather than focusing on the interests of others when making decisions; however, this tendency is sometimes influenced by social norms [98,99]. Thus, when anticipated pride was not induced, individuals showed a strong commitment of cognitive resources to green products related to self-interest. In contrast, anticipated pride could make individuals aware of their responsibility for positive social outcomes [40,41,42], making them consider social norms and how socially praiseworthy their behavior was [100]. In other words, anticipated pride raised individuals’ concerns about altruism [42,46,70]. Anticipated pride thus deflected the individual’s own egoistic tendencies, causing them to devote equal levels of cognitive resources to themselves and their environmental interests.

It is worth noting that in the P3 component, the impact of anticipated pride and message framing was not observed as regulating the influence of the type of green product on green consumption behavior. However, this did not validate H2a and H2b. Previous studies have found that the presence of the N400 component may counteract the effects of the pre-P3 component [73,101]. Thus, the failure to verify the hypotheses may be due to the fact that the inclusion of the variable of anticipated pride delayed the entire time course of individual decision processing, and the moderating effect of anticipated pride on green product type and message framing occurred at a later stage for the N400 component and LPP components.

### 4.3. The N400 and LPP Are Modulated by Green Product Type, Message Framing, and Anticipated Pride

Consistent with H3a, H3b, H4a, and H4b, it was observed that individuals in the control group had a larger N400 and a smaller LPP when deciding on other-interested green products, as opposed to self-interested green products, under loss framing. Thus, a non-significant but marginally smaller trend N400 and a larger LPP were observed in the anticipated pride group.

N400 is recognized to be relevant to cognitive and emotional conflicts in social decision-making [61,62,63]. When individuals encounter situations that violate group social norms [72] or sentences that violate their value systems [73], they experience cognitive and emotional conflicts that trigger larger N400 amplitudes. LPP represents the motivational significance of emotional stimuli [65] and on individuals’ emotional and motivational arousal from stimuli [66,67,68]. Previous studies have found that when results have greater motivational significance for individuals, they can cause an increase in LPP [102,103]. Thus, our results showed that, first, anticipated pride to some extent reduced individuals’ perceived affective conflict over other-interested green products in loss framing. When individuals’ anticipated pride was not induced, emphasizing that “not buying other-interested green products will harm the environment”, this triggered a stronger social norm violation and cognitive and emotional conflict than “not buying self-interested green products will harm physical health.” After inducing individuals’ anticipated pride, “not purchasing other-interested green products that harm environmental interests makes you feeling not pride” had a weaker sense of value system violation than “not purchasing self-interested green products that harm physical health makes you feeling not pride.” In concordance with previous findings, in the control group, the purchase motivation for other-interested green products was more in line with social expectations than the purchase motivation for self-interested green products was [44]. Therefore, facing environmental losses generated stronger social norm conflicts than facing self-interest damage [10], resulting in a larger N400 amplitude. In terms of anticipated pride, this was consistent with the results of previous research, as anticipated pride is a self-conscious emotional experience generated by individuals who engage in socially valuable or socially approved behaviors [34]. The motivation for other-interested green products was more purely altruistic than that for self-interested green products, and was more in line with social approval [44]. Therefore, when anticipated pride was paired with other-interested green products rather than self-interested green products, it was more in line with the individuals’ understanding of the conditions under which pride was generated [42,46,70,71]. That is to say, it was more logical in cognition and had a smaller sense of “dissonance” and cognitive–emotional conflicts, resulting in a trend of smaller N400 amplitude.

Second, the findings of this study demonstrated that participants had a motivational bias toward self-interest loss, and that anticipated pride could cause them to shift their motivational focus to the altruistic motivation of environmental benefit loss. Specifically, in loss framing, individuals who did not have anticipated pride induced were more motivated to focus on self-interested green products than on other-interested green products. Conversely, when anticipated pride was induced, individuals were more motivated to focus on other-interested green products than on self-interested green products. This confirmed the results of previous studies, as described below. There was a self-interest bias in the LPP component, which meant that individuals had a high intensity of arousal to self-interest losses, as reflected in the amplitude of the LPP [67,74]. From an evolutionary perspective, to survive and adapt to the environment, individuals needed to be able to quickly allocate extra attention to potential aversive stimuli and invest stronger motivational attention to those that posed a threat to themselves [73,104]. Therefore, individuals had a stronger motivation to pay attention to the loss of physical health. In contrast, anticipated pride increased individuals’ motivational concern for altruism [42,46,70] and social value [39,105]. Thus, anticipated pride reversed the original self-interest loss concern bias and made individuals more concerned about environmental benefit loss. This also validated the cognitive–affective dual-system model to some extent. Although the message framing at the cognitive level and the anticipated pride at the emotional level have not shown a joint impact on the actual purchase behavior of different types of green products (see Section 4.1 for details), they do collectively affect the late processing of individual decision-making.

Finally, this study induced a positive feeling similar to positive mood by manipulating the continuous anticipated pride emotions priming, and found that this positive feeling can help people reduce cognitive conflicts when facing environmental dilemmas, and invest more cognitive resources in the later stages of decision-making. This will further affect environmental behavior by promoting green purchasing. Consistent with previous research [96,97], this may indicate that positive mood can have a positive impact on behavior in the early and late stages of decision-making, and whether this conclusion can be achieved requires direct evidence from future research.

### 4.4. Implications and Future Directions

The findings of this study have several implications for theory. First, the obtained results underline the neural mechanisms through which green product types influence green consumer behavior under cognitive (message framing) and emotional (anticipated pride) marketing measures. Anticipated pride was found to reduce cognitive conflict and increase motivational attention to other-interested green products during loss framing. These findings broaden our understanding of green consumer behavior. Second, this study provides insights into the actual green purchasing behavior of consumers in the marketplace. When it comes to facilitating an individual’s actual purchasing behavior, whether the green product was categorized as being self-interested or other-interested, the best results were achieved when using indirect manipulation of positive moods such as anticipated pride.

It should be noted that this study has several limitations. First, while the results of this study found a tendency for actual green purchasing behavior to be facilitated when anticipated pride was combined with a gain framing for behavioral outcomes, it did not find different effects for different types of green products. Anticipated pride and message framing could only influence neural mechanisms in individuals’ decision-making for different types of green products, as shown in the late N400 and LPP components. This suggests that more effective measures must be explored that can promote actual purchase behavior for self- and other-interested green products. Second, the experimental manipulation of this study is not rigorous enough. The study compared the anticipated pride condition (anticipated pride: “I will be proud” vs. “I will not be proud”) to a blank screen in the control condition, which did not control the additional variable of sensory features (e.g., frequency of words, length of sentence). Subsequent research could improve by setting a control condition with similar word counts based on this paradigm. Third, in research, the selection of green products, especially food, is mostly vegetarian (e.g., organic apples, organic vegetables, etc.), so vegetarians may have special EEG responses to this. However, this study did not measure and control whether the participants were vegetarians, which may have an impact. In addition, the green product pictures selected in this study have bright colors, which may also affect the EEG results of the participants. These should be noted when using this paradigm in subsequent research. Fourth, this study noted the presence of the “intention–behavior” gap that has been observed in previous studies [11,12,13], but only examined actual behavior and did not measure individuals’ willingness to engage in green consumption. Subsequent research could simultaneously measure individuals’ willingness and behavior to make green purchases to better assess the existence and manifestation of this phenomenon. Fifth, the cultural differences between China and the West may have affected the experimental results. This study was conducted using Chinese students. China tends to emphasize a collectivist culture, while the West places more of a focus on individualism [106]. Therefore, a follow-up study could be conducted cross-culturally to determine whether these cultural considerations produce meaningful differences in the results. Finally, as discussed in the discussion section, this study only indirectly investigates positive moods by continuously priming anticipated pride emotions, and further research can directly manipulate positive mood to see if the two are similar.

## 5. Conclusions

This study examined the impacts of green product type, message framing, and anticipated pride on green purchase behavior, employing an ERP methodology. The primary discoveries of this study are as described below. First, anticipated pride may facilitate consumers’ green purchasing behavior. Second, anticipated pride can increase individuals’ cognitive resource investment to other-interested green products. Third, anticipated pride reduces cognitive conflict and increases motivational attention to other-interested green products under loss framing.

## Data Availability

The data presented in this study are available on request from the corresponding author. The data are not publicly available, due to concerns about privacy and ethics in personal decision-making.

## Supplementary Materials

The following supporting information can be downloaded at: https://www.mdpi.com/article/10.3390/brainsci13101427/s1, Table S1: The numbers of valid trials in each condition; Table S2: Pre-experimental evaluation of green product types; Table S3: Effect of P260 components.
