# Supplementary material for: The Influence of Green Product Type, Message Framing, and Anticipated Pride on Green Consumption Behavior: An Event-Related Potential (ERP) Study
Source: Brain Sci. 2023 Oct 7;13(10):1427. doi: 10.3390/brainsci13101427 (PMC10604928; doi:10.3390/brainsci13101427)
Supplement: Supplementary file 1 [file brainsci-13-01427-s001.zip › brainsci-2586079-supplementary.pdf]

Supplementary Materials

1.1 Numbers of valid trials in each condition

Table S1. The numbers of valid trials in each condition.

Participants	Anticipated pride group				Control group			
	Gain-self	Gain-other	Loss-self	Loss-other	Gain-self	Gain-other	Loss-self	Loss-other
[1]	61	61	61	63	56	62	58	62
[2]	63	63	63	63	57	63	56	56
[3]	59	55	59	55	61	49	61	49
[4]	59	55	59	57	51	48	58	49
[5]	63	63	62	63	62	62	63	63
[6]	62	63	61	61	57	52	59	57
[7]	62	61	60	63	56	56	51	58
[8]	58	61	59	61	60	60	59	62
[9]	58	61	51	60	49	41	40	42
[10]	63	61	59	58	60	62	60	63
[11]	62	59	61	56	61	63	62	59
[12]	56	51	54	52	53	55	55	57
[13]	55	57	53	56	58	52	57	50
[14]	56	57	57	54	58	58	56	58
[15]	58	58	49	57	63	63	62	61
[16]	61	59	60	57	59	55	55	58
[17]	50	56	52	58	61	63	62	62
[18]	54	58	55	54	63	62	62	61
[19]	63	53	63	51	61	58	63	56
[20]	57	58	59	53	55	59	53	56
[21]	57	58	57	56	61	61	63	63
[22]	57	48	55	55	59	64	52	59
[23]	60	59	56	62	46	52	51	52
[24]	61	60	58	61	45	56	51	56
[25]	52	54	50	56	59	61	57	58
[26]	59	55	54	58	63	62	60	60
[27]	57	62	55	59	62	62	57	60
[28]	55	62	54	61	56	58	53	56
[29]	50	61	45	57	52	56	55	55
[30]	55	60	53	49	59	60	58	59

1.2 Manipulation of Green products

In order to verify that the nine self-interested and nine other-interested green products in the experimental materials could effectively make participants perceive, data from 30 participants were collected on the Wenjuanxing website (https://www.wjx.cn/) for preliminary experiments. Randomize the names and image sequences of eighteen green products, and ask participants to answer the following question with a 9-point score after reading and watching the products: “Do you think this green product is more beneficial to yourself or the environment?” (1 = very beneficial to oneself; 9 = very beneficial to the environment) (Zhang et al., 2022). Then, we performed a single sample t-test for the question scores under the green products and the "neutral" value (5) of the rating scale respectively. All results were shown in Table A1 (Zhang et al., 2022). The results indicated that nine types of self-interested green products and nine types of other-interested green products were effective representatives for manipulating different types of green products.

Table S2. Pre-experimental evaluation of green product types (Zhang et al., 2022)

**Green product type**	**Products Name**	***M*±*SD***	***t***
Self-interested	1 Natural botanical shower gel	3.90±2.14	-2.817**
	2 Organic apple	3.43±2.36	-3.638***
	3 Natural vegetable soap	3.80±2.17	-3.026**
	4 Organic vegetables	3.00±2.17	-5.058***
	5 Natural organic cotton clothing	2.67±1.73	-7.393***
	6 Green rice	2.77±2.16	-5.662***
	7 Pure plant natural toothpaste	3.07±2.10	-5.043***
	8 Green wheat bread	3.10±1.94	-5.375***
	9 Natural organic cotton sheets	3.57±2.42	-3.249**
Other-interested	1 Recycled pulp flower pot	7.87±0.97	16.134***
	2 Eco-friendly gift box	7.13±1.46	8.026***
	3 Degradable plastic cup	8.47±0.78	24.466***
	4 Recycled paper	6.77±2.11	4.581***
	5 Degradable garbage bags	8.13±0.97	17.635***
	6 Environmentally friendly mobile phone holder	6.70±1.84	5.057***
	7 Degradable eco-friendly paper towels	7.53±1.41	9.857***
	8 Recycled environmental umbrellas	7.17±1.56	7.629***
	9 Green battery	7.60±1.57	9.089***

Note：** p<0.01，*** p<0.001

1.3 P260 results (210–280 ms)

According to previous studies and the topographical distribution, the P260 was calculated across ten electrode sites (CP1, CP3, CPz, CP2, CP4, P1, P3, Pz, P2, P4) in the central-parietal region (Zhang et al., 2022). The P260 amplitude was analyzed using a three-way repeated-measures analysis of variance (ANOVA) of 2 (Green product types: self-interested vs. other-interested) × 2 (Message framing: gain vs. loss) × 2 (Anticipated pride: anticipated pride group vs. control group).

Table S3. Effect of P260 components

	P260
ANOVA (F, *p*)	
green product types	2.37, *0.129*
message framing	0.03, *0.860*
anticipated pride	0.88, *0.352*
green product types × message framing	0.27, *0.608*
green product types × anticipated pride	0.10, *0.748*
message framing × anticipated pride	0.29, *0.590*
green product types × message framing × anticipated pride	0.01, *0.924*

Reference:

Zhang, G., Li, M., Li, J., Tan, M., Li, H., & Zhong, Y. (2022). Green Product Types Modulate Green Consumption in the Gain and Loss Framings: An Event-Related Potential Study. *International Journal of Environmental Research and Public Health, 19*(17). doi:10.3390/ijerph191710746
